# Electrocardiographic modifications induced by breast implants

**DOI:** 10.1002/clc.23174

**Published:** 2019-03-28

**Authors:** Sok‐Sithikun Bun, Philippe Taghji, Abdelkarim Errahmouni, Decebal Gabriel Laţcu, Alaa Al Amoura, Bogdan Enache, Thomas Hugues, Khelil Yaïci, Nadir Saoudi

**Affiliations:** ^1^ Department of Cardiology Princess Grace Hospital Monaco Monaco (Principality); ^2^ Department of Cardiology Private Clinic la Casamance Aubagne France; ^3^ Department of Cardiology Dupuytren University Hospital Limoges France; ^4^ Department of Cardiology Centre Hospitalier de Troyes Troyes France

**Keywords:** breasts implants, ECG modifications

## Abstract

**Background:**

Echocardiography realization can be challenging in the presence of breasts implants (BI). It is less known if electrocardiograms (ECG) may be modified in the presence of BI.

**Methods:**

ECG from women with BI (and without any known cardiac structural disease) were sent and analyzed by two experienced electrophysiologists (EP1 and EP2) who were blinded and completely unaware of the context of the patients (Group 1). ECG from a control matched‐group of female women without BI (Group 2) were also blindly sent for analysis.

**Results:**

ECG were collected from 28 women with BI (42 ± 8 years) without any acute medical condition. A proportion of 42% of the ECG were considered abnormal by EP1 and 46% by EP2. The abnormalities were for EP1: negative T waves (5), ST depression in inferolateral leads (2), absence of R wave progression from V1 to V4 (4), left ventricular (LV) hypertrophy (1), long QT(1), early repolarization (1), short PR (1); For EP2: negative T waves (6), ST depression in inferolateral leads (2), absence of R wave progression from V1 to V4 (4), LV hypertrophy(3), long QT (1), early repolarization (1). ECG from group 2 were considered abnormal in only 1 patient (5%) for EP1, and normal in all for EP2 (*P* = 0.0002 between the groups).

**Conclusions:**

ECG from women with BI were considered abnormal in 42% to 46% of the cases by expert readers. ECG interpretation can thus be misleading in these women.

**ABBREVIATIONS**
BI: breasts implants
ECG: electrocardiogram/electrocardiographic
EP: electrophysiologist
SHD: structural heart disease

## 1 INTRODUCTION

Different anatomical chest variations/structures are responsible for electrocardiographic (ECG) modifications in patients without heart disease. Chronic obstructive pulmonary disease is often associated with a decrease in voltage amplitude in all the ECG leads. Other anatomic variations like pectus excavatum,^1^ or situs inversus^2^ may induce ECG modifications.

Doing an echocardiography may be challenging in the presence of breasts implants (BI), as the ultrasound transmissions are impaired by the protheses structure.^3^

It is less known if an ECG may be modified by the presence of BI.

## 2 METHODS

### 2.1 Patient selection

Twelve‐lead ECGs obtained from women with BI were sent and analyzed by two experienced electrophysiologists (EP1 and EP2) who were blinded and completely unaware of the context (Group 1). None of the women had BI because of reconstructive surgery (breast cancer). The women gave their consent for their ECG to be collected for the purpose of the study. ECGs from a control group of women without BI (Group 2, n = 20) were also randomly and blindly sent for analysis. The control group included women from our nurse and paramedical staff. Exclusion criteria were: age > 55, any cardiovascular sign/disease (hypertension, stroke or congestive heart failure, diabetes, and dyslipidemia as defined by low‐density lipoprotein [LDL]‐cholesterol ≥160 mg/dL if age comprised between 20 and 39 years; or LDL‐cholesterol ≥70 mg/dL if age above 40 years). All the ECGs were exclusively performed by the nursing staff of our department and special care was taken to place the electrodes in a correct and reproducible position, despite the presence of BI (Group 1): fourth intercostal space on the right (V1) and left (V2) border of the sternum, V4 on the fifth intercostal space on the midclavicular line, V3 midway between V2 and V4, V5 on the anterior axillary line on the same horizontal level as V4, and V6 on the mid‐axillary line on the same horizontal level as V4 and V5.

All the women from both groups had an echocardiography to check for any structural heart disease (SHD) that could likely explain their ECG modification.

The electrophysiologists were asked to report the abnormalities using either the Novacode or Minnesota code as follow: left ventricular (LV) hypertrophy without ST‐T abnormalities (Novacode 6.1.0) measured with the Sokolow‐Lyon index; negative T waves were considered significant if associated with a negative phase at least 1.0 mm, but not as deep as 5.0 mm (Minnesota code 5‐2). ST depression in inferolateral leads was considered significant if comprised between 1.0 and 2.0 mm with ST segment horizontal or downward sloping (Minnesota code 4‐1‐2); absence of R wave progression (Minnesota code 1‐2‐8). The cutoff for long corrected QT (Bazetts formula) was 460 ms. Early repolarization was noted if fulfilling the last consensus conference, that is, QRS duration <120 ms, with Jp 0.1 mV in two or more contiguous leads of the 12‐lead ECG excluding V1 to V3, and presence of an end‐QRS notch or slur on the prominent R‐wave.^4^

### 2.2 Statistical analysis

The statistical analysis was completed using GraphPad Prism 5 (San Diego, California). Numerical variables are expressed as mean ± SD. A Cohens Kappa test was used for inter‐observator agreement correlation.

## 3 RESULTS

**Figure 1:**
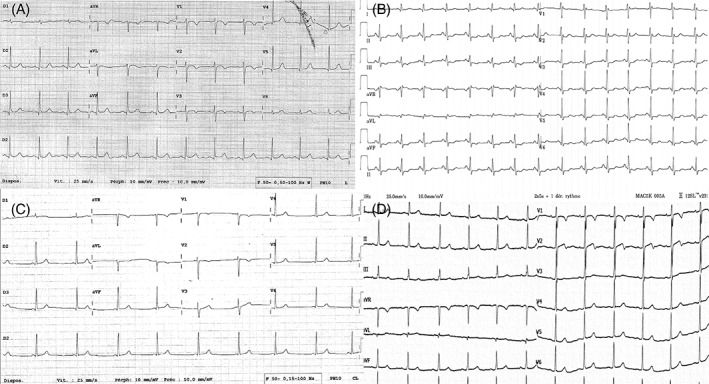
Representative cases of 12‐lead electrocardiogram modifications observed in women with breast implants and absence of structural heart disease. A, T wave inversion from V1 to V3 (patient no. 1 from the table). B, Diffuse ST depression from V3 to V6 and in inferior leads (patient no. 9). C, Short PR interval and negative T waves in V1 V2 (patient no. 21). D, Negative T waves in V1 and V2 in a 36‐year‐old patient of African origin who experienced fainting (patient no. 28)

ECGs were collected from 28 women with BI (mean age 42 ± 8 years; all of Caucasian origin except for one woman, who was from African origin). The mean time between the BI insertion and the ECG recording was 3.1 ± 2.4 years. Only one woman had an ECG before and after the insertion of her BI. None of the women had a personal history of SHD or known cardiovascular risk factors in the BI group, neither in the control group. There were no differences concerning the body mass index between the two groups (20.2 ± 5.8 in group 1 vs 22.9 ± 3.0 in group 2; *P* = 0.42). A proportion of 42% (12/28) of the ECGs was considered abnormal by EP1 and 46% (13/28) by EP2. The abnormalities (Table 1) were for EP1: negative T waves (5) (Figure 1A), ST depression in inferolateral leads (2) (Figure 1B), absence of R wave progression from V1 to V4 (4), LV hypertrophy (1), long QT (1), early repolarization (1), short PR (1) (Figure 1C).

**Table 1:** ECG analysis of patients with breasts implants

	Age	Electrophysiologist 1	Electrophysiologist 2
1	42	Negative T waves from V1 to V4, absence of R wave progression from V1 to V4	Negative T waves from V1 to V4, absence of R wave progression from V1 to V4
2	52	ST depression from V3 to V6	ST depression from V3 to V6
3	40	Early repolarization in inferior leads	Early repolarization in inferior leads
4	42	Negative T waves from V1 to V4, absence of R wave progression from V1 to V4	Negative T waves from V1 to V4, absence of R wave progression from V1 to V4
5	46	Long QT (QTc = 480 ms)	Long QT (QTc = 500 ms)
6	36	Abnormal R transition from V1 to V4	Left ventricular hypertrophy (Sokolow = 36 mm)
7	57	Normal	Normal
8	46	Normal	Normal
9	43	ST depression in inferior leads	ST depression in inferior leads
10	39	Absence of R wave progression from V1 to V4	Left ventricular hypertrophy (Sokolow = 38 mm)
11	31	Normal	Normal
12	26	Normal	Negative T waves from V1 to V3, absence of R wave progression from V1 to V4
13	42	Normal	Normal
14	25	Normal	Normal
15	30	Normal	Normal
16	48	Normal	Normal
17	58	Normal	Normal
18	34	Left ventricular hypertrophy (Sokolow = 35 mm)	Left ventricular hypertrophy (Sokolow = 35 mm)
19	50	Normal	Normal
20	36	Normal	Normal
21	23	Negative T waves V1‐V2/Short PR	Negative T waves V1‐V2, absence of R wave progression from V1 to V4
22	42	Normal	Normal
23	51	Normal	Normal
24	41	Normal	Normal
25	39	Normal	Normal
26	38	Normal	Normal
27	42	Negative T waves in D3 VF	Negative T waves in D3 VF
28	36	Negative T waves in V1 and V2	Negative T waves in V1 and V2

For EP2, the abnormalities were: negative T waves (6), ST depression in inferolateral leads (2), absence of R wave progression from V1 to V4 (4), LV hypertrophy (3), long QT (1), early repolarization (1).

The patient with a possible diagnosis of long QT had an adrenalin challenge to rule out any long QT syndrome; the patient with short PR did not elicit any preexcitation after an adenosine injection. A normal echocardiography also excluded any LV hypertrophy by the measurement of the interventricular septum diameter.

The inter‐observator agreement was calculated at 92.3%.

ECGs from group 2 (38 ± 7 years, all of Caucasian origin) were considered abnormal only in one woman of group 2 (5%) for EP1 (absence of R wave progression from V1 to V4), and all normal for EP2. The inter‐observator agreement was calculated at 96.4% in the control group.

## 4 DISCUSSION

The present study shows a significantly higher proportion of ECG modifications in women with BI and no SHD, when compared to women without BI. The implications may be important, because ECG interpretation can be completely misleading if the patient presents with a cardiovascular symptom/sign.

A 42% to 46% of ECG modifications were reported in our study, which is significantly higher than the prevalence of ECG abnormalities reported in a very large population of non‐athlete young female subjects, including a vast majority of Caucasian ethnicity (7764 women), mainly involving QT abnormalities.^5^ This is in contrast with our findings (in almost only Caucasian young female after BI), which revealed ECG modifications such as negative T waves, ST depression in inferolateral leads or absence of R wave progression from V1 to V4 (abnormal QT interval accounted for less 4% of the cases in our study).

Of note, in our study, the presence of BI affected the depolarization as well as the repolarization on the different ECGs analyzed. The ECG modifications were also more predominantly observed on the precordial (chest) leads, in comparison to the limb leads, which is more likely to be explained by the presence of the BI. In the BI group, two patients showed a short PR and long QT interval (PR and QT measured in lead II), respectively. These borderline intervals may represent the normal variations in the general population, and do not seem related to the BI themselves.^6^ T wave inversion and ST depression have sometimes been reported as nonspecific modifications in women, but they were considered “abnormal” enough for the two expert electrophysiologists to be reported.^7^, ^8^ Furthermore, the higher proportion of these ECG modifications is significant in comparison with the control group (none in the later).

Figure 1D illustrates the case of a 36‐year‐old patient who was admitted for several episodes of fainting. She had no significant past medical history (but implanted with BI), and of African origin. There was a family history of unexplained sudden cardiac death (the father and the brother of the patient, respectively at the ages of 50 and 45). Despite normal morphological evaluation including echocardiography and cardiac MRI, we decided to insert an implantable loop recorder (Reveal Linq, Medtronic Inc, Minneapolis, Minnesota, USA.) in this patient because of the ECG « abnormalities ». Those ECG modifications were finally attributed to the presence of BI in this patient as no arrhythmia was recorded after 1 year of follow‐up with the implantable loop recorder, despite the recurrence of a vasovagal syncope.

Literature on this subject is very limited. Previous work by Lu et al who reported normal ECGs in 10 out of 11 patients with silicone BI and all complaining of atypical chest pain.^9^ All patients had the BI removed and the authors concluded that silicone BI might induce atypical chest pain related to local inflammatory reactions.

ECG modifications in women with BI may be explained by two mechanisms. The first one could be incorrect electrode placement (Appendix S1, Supporting Information). A significant volume of the BI may make the positioning of the electrodes more difficult in clinical practice (V1 to V3 may be more concerned for the positioning of the electrodes in the presence of BI). Peters et al reported a case of myocardial infarction mimicked by misplacement of the V2 and V3 leads because of a severe capsular contracture. The ECG normalized after an open capsulotomy on this patient.^10^

**Figure 2:**
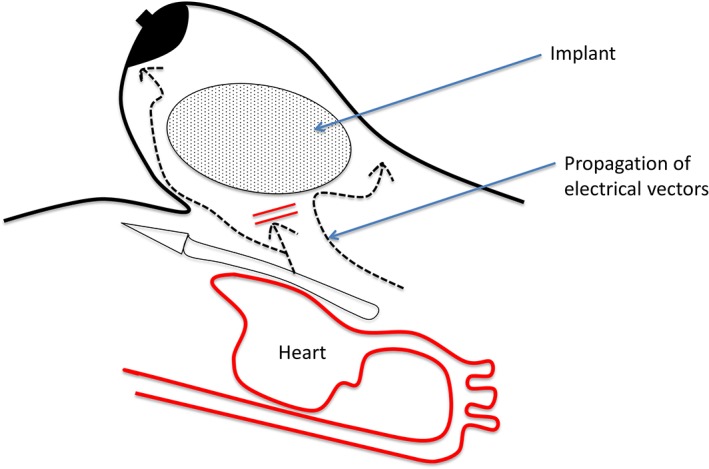
Scheme explaining the deviation of the electrical wave front due to the presence of the breast implants acting as an unexcitable barrier

One possible hypothesis could be electrical vector deviations emanating from the heart, because the different wave fronts encounter an unexcitable region (silicone) before reaching the surface of the skin (Figure 2). This hypothesis is speculative, and needs be confirmed by experimental studies, but is an extension of the phenomenon observed with ultrasounds propagation in the presence of BI when performing an echocardiography.

It may be a reasonable advice to suggest to women who are planning to have BI insertion to have an ECG before and keep it in their file to serve as a comparison for the future, in case of the appearance of any cardiovascular symptoms, in their medical history.

### 4.1 Limitations

This is a monocentric study. The number of women with ECGs before the insertion of BI is limited (n = 1). This is due to the fact that ECGs are not systematically recommended as part of the preoperative (anesthesiologist) visits in young women without SHD nor cardiovascular risk factors. A prospective study is needed, aiming to compare ECGs before BI insertion with post‐operative ECG.

The data on the size of the BI in our population was not available to assess a possible correlation between the size of the BI and ECG modifications.

## 5 CONCLUSION

ECGs obtained from women with breasts implants were considered abnormal in 42% to 46% of the cases in comparison with a control group of women without breast implants (*P* = 0.0002). ECG interpretation can be misleading in the context of chest pain/acute coronary syndrome occurring in these patients.

## CONFLICT OF INTEREST

The authors declare no potential conflict of interests.

## Supporting information


**Appendix S1.** Variations of the placement of the electrodes that may exaggerate the electrocardiographic modifications in the presence of breast implants (V3 and V4 in this illustration were intentionally placed in an incorrect position).
