# Supplementary material for: Electrocardiographic modifications induced by breast implants
Source: Clin Cardiol. 2019 Mar 28;42(5):542–5. doi: 10.1002/clc.23174 (PMC6522987; doi:10.1002/clc.23174)

**Supplementary materials.** Variations of the placement of the electrodes that may exaggerate the electrocardiographic modifications in the presence of breast implants (V3 and V4 in this illustration were intentionally placed in an incorrect position).


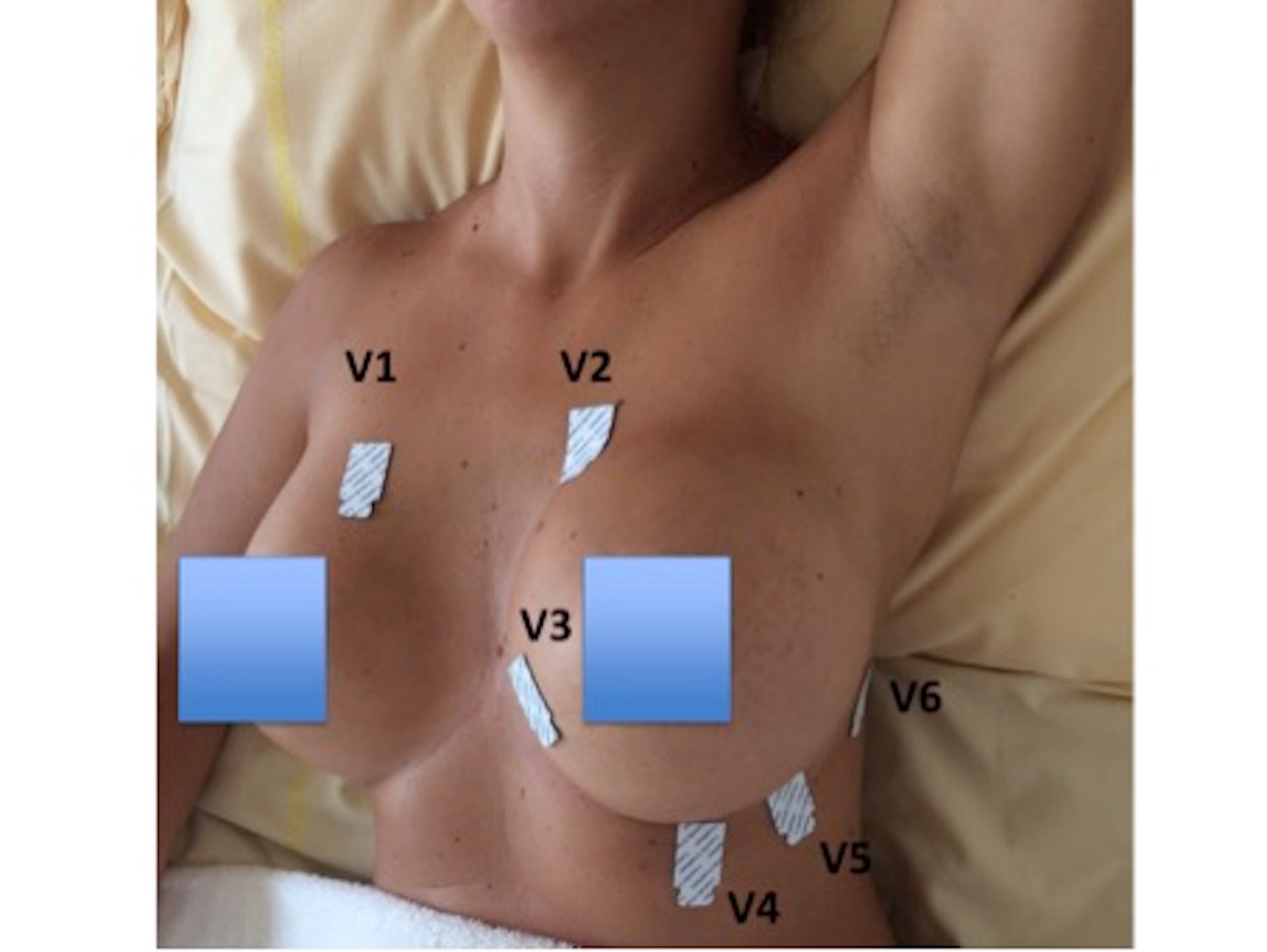

Supplement: Supplementary file 1 — Appendix S1. Variations of the placement of the electrodes that may exaggerate the electrocardiographic modifications in the presence of breast implants (V3 and V4 in this illustration were intentionally placed in an incorrect position). [file CLC-42-542-s001.docx]
